# Invariance properties of bacterial random walks in complex structures

**DOI:** 10.1038/s41467-019-10455-y

**Published:** 2019-06-04

**Authors:** Giacomo Frangipane, Gaszton Vizsnyiczai, Claudio Maggi, Romolo Savo, Alfredo Sciortino, Sylvain Gigan, Roberto Di Leonardo

**Affiliations:** 1 Dipartimento di Fisica, Sapienza Università di Roma, Piazzale A. Moro 5, I-00185 Roma, Italy; 2 NANOTEC-CNR, Soft and Living Matter Laboratory, Institute of Nanotechnology, Piazzale A. Moro 5, I-00185 Roma, Italy; 3 Laboratoire Kastler Brossel, Sorbonne Université, Ecole Normale Supèrieure, Collège de France, CNRS UMR 8552, 24 rue Lhomond, 75005 Paris, France; 4 Optical Nanomaterial Group, Department of Physics, Institute for Quantum Electronics, ETH Zurich, Auguste Piccard Hof 1, 8093 Zurich, Switzerland

**Keywords:** Cellular motility, Colloids, Biological physics

## Abstract

Motile cells often explore natural environments characterized by a high degree of structural complexity. Moreover cell motility is also intrinsically noisy due to spontaneous random reorientations and speed fluctuations. This interplay of internal and external noise sources gives rise to a complex dynamical behavior that can be strongly sensitive to details and hard to model quantitatively. In striking contrast to this general picture we show that the mean residence time of swimming bacteria inside artificial complex microstructures is quantitatively predicted by a generic invariance property of random walks. We find that while external shape and internal disorder have dramatic effects on the distributions of path lengths and residence times, the corresponding mean values are constrained by the sole free surface to perimeter ratio. As a counterintuitive consequence, bacteria escape faster from structures with higher density of obstacles due to the lower accessible surface.

## Abstract (web summary)

It has been previously shown theoretically that the average path length of random walks inside a closed domain is invariant. Here the authors demonstrate that this invariance property can be used to predict the mean residence time of swimming bacteria exploring structured micro-environments.

## Introduction

Swimming bacteria must find their way through complex structures that are so common both in their natural environment^1,2^ and also in the laboratory^3–5^. More recently, microfabrication techniques have been increasingly used to design artificial environments with precise and tunable internal structure^6–8^. For example, arrays of scattering obstacles have been used to demonstrate the possibility of rectification and sorting in self-propelled systems^9–11^. In these applications it is often found that small details in dynamical behavior can have significant quantitative consequences. For example, the timescale of spontaneous reorientation or the distribution of scattering angles produced by obstacles have a dramatic effect on the rectification efficiency of microstructures^12–14^. In striking contrast, a recently rediscovered invariance property of random walks implies that the average path length inside a closed domain of arbitrary shape is only proportional to the volume to surface ratio with a numerical prefactor that solely depends on the spatial dimensions^15,16^. Despite its wide generality, this invariance property has started to find applications only very recently, mainly in the field of light propagation in turbid media^17,18^.

Here we study motile bacteria exploring artificial microstructures with arbitrary geometries and a wide range of obstacle densities. Using the invariance property, we quantitatively predict the mean length of the paths traced by bacteria inside these quasi-2D microstructures. Assuming that the mean swimming speeds are not significantly perturbed by the presence of obstacles, we further predict and experimentally verify the invariance of the mean residence time, a more biologically relevant quantity. As a result, for all obstacle densities, the mean residence time is only determined by the microstructure surface to perimeter ratio and the mean inverse speed.

## Results

### Design of artificial complex environments

We use direct laser writing by two-photon polymerization^19,20^ to build 3D microchambers consisting of a thick “roof” supported by randomly distributed pillars fabricated on a microscope coverslip. A 1.4-μm-thick gap between the coverslip and the roof confines bacteria in a quasi-2D geometry. The entire path traced by the cells inside the microchambers is therefore restricted on the focal plane and can be easily tracked by digital video microscopy (see Supplementary Movie 1). Moreover, the presence of two close surfaces prevents circular swimming caused by hydrodynamic coupling to a single solid wall^21,22^. In all experiments we use a smooth-swimming strain of *E*. *coli* bacteria expressing a red fluorescent protein. Microstructures are immersed in a low-density cell suspension (~3 × 10^6^ cells ml^−1^, see Methods) that we track by epifluorescence videomicroscopy. The pillars play the role of obstacles and by varying their number we can tune the degree of internal complexity of the structures. The obstacles are fabricated as randomly distributed and non-overlapping short segments measuring 3.7 μm in length and with a thickness of 0.7 μm (see Supplementary Fig. 1). Obstacles with such an elongated shape significantly deflect colliding bacteria and can be densely packed inside microstructures.

### Mean path length invariance in structures with increasing obstacle density

**Fig. 1:**
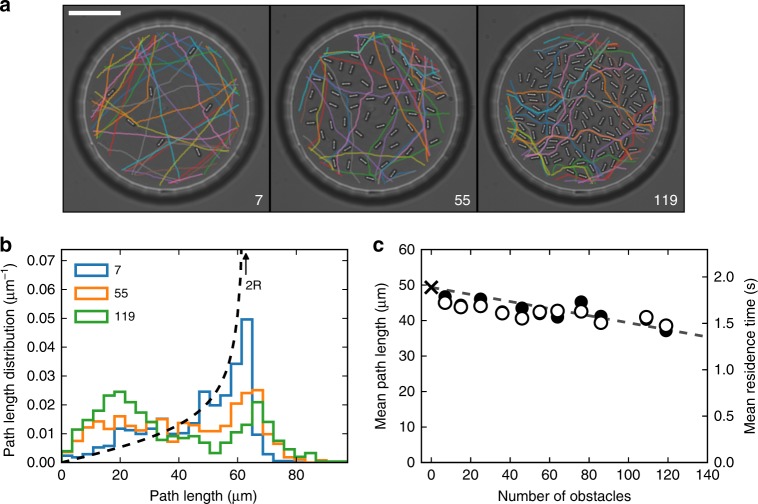
Invariance in structures with varying obstacle density. **a** Optical microscopy images of three sample microstructures with number of obstacles 7, 55, and 119. The scalebar is 20 μm. The colored lines are sample trajectories of swimming bacteria inside the micro-structures. As the number of obstacles increases, trajectories become more irregular due to scattering by obstacles. **b** Path length distributions (colored bar plot) for the structures displayed in **a**. The dashed line is the theoretical prediction in the absence of obstacles. **c** Full symbols (left *y*-axis) and open symbols (right *y*-axis) represent respectively the mean path length and the mean residence time as a function of the number of obstacles. The cross is the prediction of the invariance theorem in the absence of obstacles. The dashed line is a linear fit with the modified formula that accounts for the excluded surface due to obstacles (Eq. 4)

We fabricate a total number of 11 circular structures with a radius *R* = 31 μm and with a varying number of obstacles *N* = 7, 15, 25, 36, 46, 55, 64, 76, 86, 109, and 119. When *N* is small, bacteria traverse the structures with approximately straight trajectories (see Fig. 1a). Since the flux of bacteria through a line element of the boundary is proportional to the cosine of the angle between the cell swimming direction and the local boundary normal, the distribution of path lengths *L* for straight trajectories is given by the chord-length distribution $p(L) = L/\left( {2R\sqrt {4R^2 - L^2} } \right)$. As shown in Fig. 1b, this distribution increases monotonically diverging at the maximum value *L* = 2*R*. The experimental distribution of path lengths, obtained by tracking ~1000 bacteria in a structure with *N* = 7 obstacles, closely follows the theoretical distribution with small deviations due to rotational diffusion of bacteria and to scattering by the few isolated obstacles. When the number of obstacles is increased, bacteria are more likely to be back-scattered by obstacles close to the domain boundary giving rise to an excess of short paths that appears as a growing peak at small path lengths. At the same time, dense obstacles produce multiple deflections that result in trajectories with a total length that can exceed the circle diameter producing a tail in the distribution at *L* > 2*R*.

Despite the strong dependence on *N* of the path length distribution, the invariance theorem can be used to obtain a strikingly simple formula for the mean path length that quantitatively matches observed values for all *N*. To this aim we now illustrate a derivation of the invariance theorem using an ergodic type argument^15,23^. Let’s imagine to observe for a total time *T* the motion of *n* non-interacting random walkers moving with constant speed *v* over a region of space of total area ${\mathrm{\Sigma }}$. If reorientation dynamics is isotropic, the walkers will fill the region with a uniform concentration $\rho = n/{\mathrm{\Sigma }}$ and an everywhere isotropic velocity distribution. The time *t* spent by each walker inside a generic subregion of area *S* will be given by:

1 $$t = T\frac{S}{{\mathrm{\Sigma }}}$$

Alternatively we can obtain *t* as the mean residence time $\tau$ multiplied by the number of times each walker is expected to enter the subregion during the observation time *T*. This number can be obtained by counting the number of bacteria that enter the subregion in a total time *T* and then divide by *n*. Since, for an isotropic velocity distribution, the inward flux of walkers is $\rho v$/*π* over the entire perimeter *P* of the subregion, we have:

2 $$t = \tau \frac{{\rho v}}{\pi }P{\kern 1pt} T\frac{1}{n}$$

Equating (1) and (2) we obtain:

3 $$\ell = v\tau = \pi \frac{S}{P}$$

When the subregion contains rigid obstacles, the above derivation remains valid as long as their presence does not significantly perturb the homogeneity of the density field in the accessible space and the isotropy of random walks along the domain boundary^24^. This condition is fulfilled for small and convex obstacles producing scattering events lasting for a short interaction time. In this regime, however, the total area *S* in Eq. 3 must be replaced with the total accessible area $S\prime = S - Ns$ where *s* is the excluded area due to a single obstacle and *Ns* the total excluded area. Therefore the mean path length $\ell$ is expected to decrease linearly with the number of obstacles according to the law:

4 $$\ell = \pi \frac{S}{P}\left( {1 - \frac{{Ns}}{S}} \right)$$

In Fig. 1c we report as solid circles the experimental values of the mean path lengths as a function of the number of obstacles. Experimental data closely match the predicted linear behavior in (Eq. 4) with an intercept fixed at *πS*/*P* = *πR*/2 = 49.3 μm and a value for *s* = 6.3 μm^2^ that is independently estimated by dilating the physical boundary of the obstacle by half the cell width (0.4 μm^25^) (see Supplementary Fig. 1).

### Mean residence time

There are many situations in which the residence time is more relevant then the path length. For example, if adhesion to surfaces is an activated process^26^ the probability that a cell will remain stuck inside a structure will be proportional to the total time spent inside it. The population average residence time *τ* can be related to the mean path length under the reasonable assumption that cell speeds *v* are uncorrelated to path lengths *L* so that:

5 $$\tau = \langle L/v\rangle = \langle L\rangle {\kern 1pt} \langle v^{ - 1}\rangle = \ell /\bar v$$

with $\bar v = 1/\langle v^{ - 1}\rangle$. Although speeds can be broadly distributed in a cell population we can again use the invariance property to quantitatively predict mean residence times once the characteristic speed $\bar v$ is known. The measured mean residence time as a function of *N* is plotted in Fig. 1c showing again a close agreement with the theoretical prediction in Eq. 5 where the parameter $\bar v$ has been independently measured from experimental trajectories.

### The effect of size and shape

We have demonstrated that, for a fixed shape, the density of obstacles has a strong effect on the distribution of path lengths while leaving the mean value unchanged as long as the total internal accessible area remains the same.

**Fig. 2:**
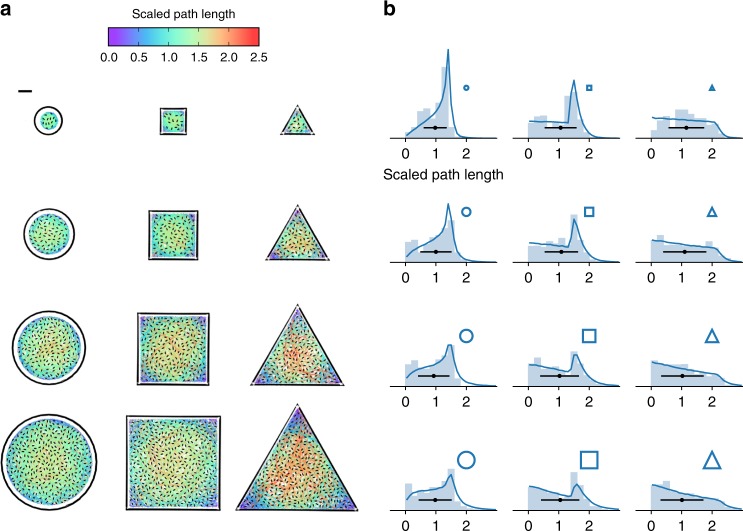
Size and shape effects on path length distributions. **a** We fabricate microstructures with circular, squared and triangular shapes all in four different sizes varying by an overall factor of 4 (the scalebar is 20 μm). The density of obstacles is the same for all structures. Bacterial trajectories are plotted over microstructure images that have been processed for contrast enhancement. Trajectories are color coded according to their scaled length (see colorbar). **b** Bar plots are the experimental distributions of scaled path lengths for each of the structures in **a**. The black points represent the mean scaled path length and the errorbar the corresponding standard deviation. Lines are theoretical distributions obtained by the numerical simulation of a Lorentz gas model with anisotropic obstacles mimicking experimental conditions

**Fig. 3:**
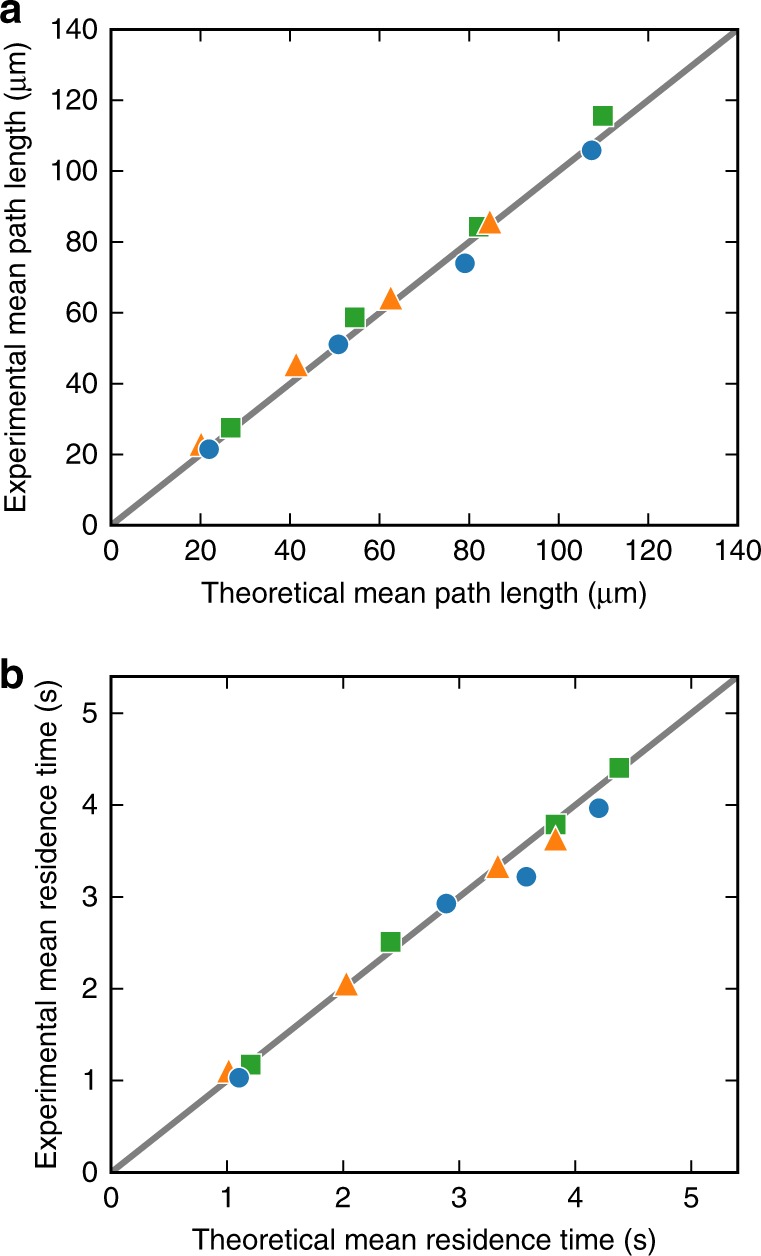
Invariance in structures with different size and shape. **a** Experimental vs theoretical mean path lengths. Each shape is represented by the corresponding symbol (circle, square, triangle) appearing four times, one for each of the four sizes in Fig. 2. All structures contains obstacles with the same density. The line passing through zero represents perfect agreement between theory and experiments. **b** Same as **a** for the mean residence time

We will now investigate the effects of shape and size on the path length distributions and again conclude that, although path distributions are very sensitive to the detailed geometry of the domain, mean path lengths and residence times can be accurately predicted using only the surface to perimeter ratio. We fabricate microstructures of circular, squared and triangular shapes all in four different sizes as shown in Fig. 2a. In all structures the obstacle density is fixed to *N*/*S* = 0.016 μm^−2^. All recorded trajectories are also plotted in Fig. 2a with a color map encoding the scaled path length *L*/$\ell$. In Fig. 2b we plot for each structure the histogram of path lengths normalized by the expected value $\ell$ in Eq. 4. As we move from circles to squares and then to triangles we observe that the corresponding histograms are characterized by larger frequencies for both short and long path lengths. The probability of short paths increases as we introduce convex corners and reduce their angle, as evidenced by the blue-violet color of the corners in squared and triangular structures. The presence of longer paths in triangles can be also understood noting that the normalized maximum straight paths for circles, square and triangles are respectively 4/*π*, $4\sqrt 2$/*π* and $4\sqrt 3$/*π* (see Supplementary Fig. 2). Despite these qualitative and quantitative differences between histograms, mean path lengths are always close to 1 when normalized by the predicted value $\ell$ as shown in Fig. 2b. The observed path length distributions can be very well reproduced by a Lorentz gas model where collisions with obstacles are described as instantaneous reorientation events^27^. These events are assumed to occur with a constant probability per unit path length given by $\rho {\kern 1pt} \sigma$, where $\sigma = 2b$/*π* is the rotational average cross section of line obstacles with length *b* (see Methods). For perfectly straight trajectories, circles and squares have path length distributions with a vertical asymptote. A strong peak is found at the location of this divergence and is progressively smeared as the linear size of the domain becomes larger than the mean free path $\lambda = (\rho \sigma )^{ - 1}$. Upon increasing the size of the region a peak around $L = 0$ appears due to bacteria that scatter close to the boundary, reorient and exit. The mix of these two types of trajectories gives rise to the observed double-peak structure. Differently for the triangle no asymptote is present in the *P*(*L*) for large *λ*, therefore only a peak at $L = 0$ progressively appears. These results show that *P*(*L*) depends strongly on the random walk properties (as the mean free path), and on the domain features (shape and size). Differently the mean values $\ell$ and $\tau$ depend only on the surface to perimeter ratio of the domain as expressed by Eq. 4. Experimental values of $\ell$ are shown Fig. 3a for all shapes and sizes. We find an excellent agreement with theory using the same value *s* = 6.3 μm^2^ that was used previously to predict the experimental values of $\ell$ as a function of the number of obstacles (see Fig. 1c). This value of *s* has also been used to calculate $\tau$ according to Eq. 5 which captures quantitatively well the experimental data as shown in Fig. 3b.

## Discussion

In conclusion, we have shown that bacterial random walks inside complex and crowded microstructures do obey a general invariance property that constraints the mean value of the path length to a simple and predictable value uniquely determined by the surface to perimeter ratio of the structure. More interestingly the mean residence time is also invariant as long as the surface to perimeter ratio remains constant. In a counterintuitive way, a greater number of obstacles does not increase the average residence time, but shortens it by decreasing the total accessible surface. Our results are very general and should remain valid also for any other type of swimming microorganisms or self-propelled colloids provided that the obstacles do not capture the particles for prolonged periods and do not alter the conditions of an isotropic velocity distribution over the domain’s boundary, as for example would happen if our obstacles were all aligned. In this direction, an interesting question could be how to generalize our results in the presence of further reorientation mechanisms, such as tumbling in wild type bacteria. The robustness of these results also provides a solid starting point for further generalizations of the invariance formula to include the effects of interactions in dense systems, the presence of flow or chemotaxis.

## Methods

### Microfabrication

The microchamber structures were fabricated with a custom built two-photon polymerization setup^28^ from SU-8 photoresist (MicroChem Corp). A single fabrication focus was used with 8 mW laser power and 100 μm s^−1^ scanning speed. After the laser fabrication scan the SU-8 photoresist sample was baked at 100 °C for 7 min, then developed by its standard developer solvent and finally rinsed in a 1:1 mixture of water and ethanol. Strong adhesion of the microchamber structures to the carrier coverglass was ensured by a layer of OmniCoat adhesion promoter (MicroChem Corp).

### Microscopy

Epifluorescence imaging were performed on an inverted optical microscope (Nikon TE-2000U) equipped with a 60× (NA = 1.27) water immersion objective and a high-sensitivity CMOS camera (Hamamatsu Orca Flash 4.0).

### Cell cultures

The *E*. *coli* strain used is the HCB437^29^, a smoooth-swimmer, transformed by a plasmid expressing the red fluorescent protein mRFP1 under the control of the lacI inducible promoter (BioBricks, BBa_J04450 coding device inserted in pSB1C3 plasmid backbone, present in the iGEM Catalogue [http://parts.igem.org/Catalogue]). Cells were grown overnight in 10 mL of LB supplemented with kanamycin (30 μg mL^−1^) and chloramphenicol (20 μg mL^−1^) in a shaking incubator at 33 °C and 200 rpm. In the morning 50 μL of the saturated culture was diluted (1:100) into 5 mL of tryptone broth supplemented with antibiotics and incubated in the same condition for 4 h (until OD_590_ ~ 0.2 is reached). The production of mRFP1 was induced by adding 1 mM IPTG, and the cultures were grown for 2 further hours until exponential phase (OD_590_ ~ 0.6–0.8). Cells were collected and washed three times by centrifugation (1500 rcf, 5′) in motility buffer (MB: 0.1 mM EDTA, 10 mM phosphate buffer, 10 mM glucose and 0.02% Tween20, pH 7).

### Simulations

We simulate bacterial trajectories as straight runs intercalated by random reorientations (run and tumble dynamics) that mimic collisions with obstacles. Run lengths are exponentially distributed with a mean free path $\lambda = (\rho \sigma )^{ - 1}$. The obstacle density $\rho$ is fixed to the experimental value *ρ* = 0.016 μm^−2^. The obstacle cross section *σ* = 2.9 μm is evaluated as the average cross section of a randomly oriented line with length *b* = 4.5 μm, i.e. $\sigma = \pi ^{ - 1}{\int} d\theta {\kern 1pt} b{\kern 1pt} {\mathrm{cos}}(\theta ) = 2b$/*π*. Assuming that an elongated particle fully aligns with a line-shaped obstacle, collisions will produce angular deflections *δ* that are only determined by the obstacle orientation and restricted to the interval [−*π*/2, *π*/2]. Weighting each outgoing direction with the cross section of the correspondingly tilted obstacle we obtain the distribution of deflection angles *p*(*δ*) = sin |*δ*| for |*δ*| ≤ *π*/2 and zero elsewhere. We also account for a small reduction in *δ* that could be caused by hydrodynamic interactions, causing particles to curve around the obstacle edges^30^, or by the fact that reorientation during collision takes a finite time^22^, while the obstacle has finite length. The total deflection angle is then set to $\delta {\prime} = \delta - {\mathrm{sign}}(\delta ){\kern 1pt} \alpha$, where *α* is set to *α* = 0.39(≈*π*/8) to fit the histograms in Fig. 2b.

### Reporting summary

Further information on research design is available in the Nature Research Reporting Summary linked to this article.

## Supplementary information

Supplementary Information

Description of Additional Supplementary Files

Supplementary Video 1

Reporting Summary

## Data Availability

The data that support the plots within this paper and other findings of this study are available from the corresponding authors upon reasonable request.

## Code availability

The codes that support the findings of this study are available from the corresponding authors upon reasonable request.
