# Supplementary material for: Invariance properties of bacterial random walks in complex structures
Source: Nat Commun. 2019 Jun 4;10:2442. doi: 10.1038/s41467-019-10455-y (PMC6547659; doi:10.1038/s41467-019-10455-y)
Supplement: Supplementary file 1 — Supplementary Information [file 41467_2019_10455_MOESM1_ESM.pdf]

Invariance properties of bacterial random walks in complex structures

G. Frangipane *et al.*

Supplementary Information

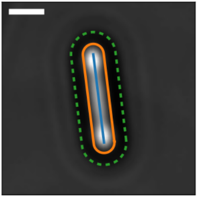

Supplementary Figure 1: Bright-field image of one single obstacle (scale bar 1 microns). The blue line is the laser scan-line during micro-fabrication. The orange line is the resulting photopolymerized volume computed by considering the laser point-spread function and the SU8 photoactivation threshold. The green dashed line represents the excluded area computed by combining the obstacle area and the minimum distance between the center of a bacterium and the obstacle (i.e. the thickness of the cell body which is 0.4 microns)

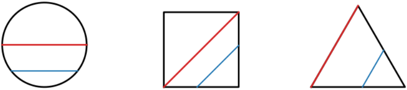

Supplementary Figure 2: Examples of straight paths with maximum length (red) to be compared with paths having average length (blue), plotted for the circular, squared and triangular domains.
