# Supplementary material for: Invariance properties of bacterial random walks in complex structures
Source: Nat Commun. 2019 Jun 4;10:2442. doi: 10.1038/s41467-019-10455-y (PMC6547659; doi:10.1038/s41467-019-10455-y)
Supplement: Supplementary file 2 — Description of Additional Supplementary Files [file 41467_2019_10455_MOESM2_ESM.pdf]

Supplementary Movie 1 Legend: Bacteria swimming in disordered microstructures with an increasing density of obstacles (speed 2x).
